# Oxytocin and emergency caesarean section in a mediumsized hospital in Pakistan: A cross-sectional study

**DOI:** 10.18332/ejm/124111

**Published:** 2020-08-06

**Authors:** Mirjam Lukasse, Ingrid Hovda, Sara Thommessen, Sosan McAuley, Marian Morrison

**Affiliations:** 1 Institute of Nursing and Health Promotion, Faculty of Health Sciences, Oslo Metropolitan University, Oslo, Norway; 2 Department of Nursing and Health Sciences, Faculty of Health and Social Sciences,University of South-Eastern Norway, Borre, Norway; 3 Women's Christian Hospital,Multan, Pakistan

**Keywords:** caesarean section, oxytocin, obstetric practice, Low-MiddleIncome countries, Pakistan

## Abstract

**INTRODUCTION:**

One of the most common complications during labor is prolonged labor (dystocia), which is associated with risks for the mother and fetus. Dystocia is usually treated with oxytocin, which is also used to induce labor. Oxytocin may not have the desired effect of progress and can negatively affect the fetus, thus resulting in an emergency caesarean section (CS). The aim of this study was to describe obstetric practice, use of oxytocin and its association with an emergency CS.

**METHODS:**

A cross-sectional retrospective register study was conducted that included all women who gave birth during 2014 and 2015 at a hospital in a large city in Pakistan.

**RESULTS:**

A total of 6652 women gave birth to 6767 newborns, 66.8% were multiparous and 33.2% primiparous women. Of the primiparous women, 78.9% had a spontaneous vaginal birth, 1.2% an elective CS and 14.4% an emergency CS. Of the multiparous women, 81.9% had a spontaneous vaginal birth, 8.0% an elective CS and 6.7% an emergency CS. Operative vaginal birth was 2.1% among primiparous and 0.2% among multiparous women. Oxytocin for induction or augmentation was administered to 60.0% of primiparous and 30.5% of multiparous women. Oxytocin during the first stage of labor was associated with an increased risk for emergency CS for both primiparous and multiparous women.

**CONCLUSIONS:**

Despite the association between oxytocin and emergency CS, the CS rate was low in this hospital. The majority of the women gave birth vaginally, even with a breech presentation. Few operative vaginal births were performed.

## INTRODUCTION

Births assisted by skilled health personnel decrease the risk of maternal and neonatal mortality^1-3^. Efforts to increase births attended by skilled health professionals often lead to birth in a health facility/hospital^3^. Attendants in health facilities can intervene in case of complications^4^.

One of the most common complications during labor, in particular in primiparous women, is prolonged labor or dystocia^5,6^. Dystocia, also defined as less progress than expected or desired during a certain period of time, is associated with a number of complications such as maternal exhaustion, fetal hypoxia and asphyxia, and postpartum hemorrhage (PPH), and very occasionally ruptured uterus^5,7,8^. Globally, dystocia is most commonly treated with the intravenous administration of oxytocin^9-11^. Besides being used for augmentation, oxytocin is commonly used for induction of labor, pre-labor rupture of membranes (PROM), and PPH^9,12-15^. Risks associated with the use of oxytocin are uterine hyper-stimulation, fetal hypoxia and asphyxia, uterine rupture and PPH^9,16,17^. Close monitoring of fetal heart rate and uterine activity by a skilled professional in a health facility is therefore recommended as long as infusion of oxytocin is ongoing^9,16,17^. In case where, despite the treatment with oxytocin, progression in labor fails, caesarean section (CS) is usually indicated^17,18^. The fetus can also react to (hyper) stimulation and become the reason for an emergency CS^9,17,19^. While oxytocin is a powerful and much used drug, there are concerns for its overuse^9,20^. The registration and monitoring of the use of interventions in obstetric care are part of essential quality control as well as registering obstetric and neonatal outcomes^19,21^.

There are numerous studies investigating obstetric care in Pakistan^4,22,23^. The rate of caesarean section, indications for CS, different methods for induction, associated factors with ruptured uterus and severe perineal trauma are among central obstetric issues investigated^4,22-25^. The majority of these studies were performed at university, public and teaching hospitals, or based on demographic health surveys^4,22,23^. We did not find any studies from Pakistan on the prevalence of intravenous oxytocin during labor and the association with emergency caesarean section (CS).

The first objective for this study was to present aspects of the obstetric care in a medium-sized, private hospital in a large city in Pakistan. The second objective was to explore the use of oxytocin and its association with emergency CS.

## METHODS

This cross-sectional study used data routinely collected at the hospital. The data included all women who gave birth to 6767 babies in 2014 and 2015 (N=6652). Maternal age, gestational age, parity and the number of antenatal visits at the study hospital were recoded from a continuous variable into categorical/dichotomous variables as shown in Table 1. Mode of delivery was recoded into five groups: spontaneous vaginal birth, operative vaginal birth, vaginal breech delivery, elective CS and emergency CS. Perineal trauma and episiotomy was recoded into: episiotomy ‘yes’ or ‘no’, and perineal trauma by degree of tear. The number of fetuses, gender at birth, fetal presentation and/or lie and their state at birth (alive, stillbirth or neonatal death) was recorded. The use of oxytocin, the indication(s) and during which stage of labor it was administered was recorded. Indications for induction were categorized after primary indication. Fetal reasons for induction included intrauterine growth restriction, little fetal movement, oligohydramnios, post maturity and fetal death. Maternal reasons included diabetes and hypertensive complications. Doctors registered women as having a bad obstetric history when there was a history of multiple previous miscarriages, long infertility, previous stillbirths, neonatal deaths and previous small for dates baby and similar. Other methods for induction and augmentation were balloon catheter and orally misoprostol alone, or in addition to oxytocin. The primary indications for CS indication were used and categorized as shown in Table 1.

**Table 1:** Background characteristics by parity on admission (N=6652)

	*Primiparous women n=2208*	*Multiparous women n=4444*	*Total N=6652*
	*n (%)*	*n (%)*	*n (%)*
**Year**			
2014	1153 (52.2)	2320 (52.2)	3473 (52.2)
2015	1055 (47.8)	2124 (47.8)	3179 (47.8)
**Maternal age** (years)			
<20	66 (3.0)	8 (0.2	74 (1.1)
20–24	956 (43.3)	777 (17.5)	1733 (26.1)
25–29	906 (41.0)	1735 (39.0)	2641 (39.7)
30–37	272 (12.3)	1752 (39.4)	2024 (30.4)
>37	8 (0.4)	172 (3.9)	180 (2.7)
**Gestational age** (weeks) (n=6648)			
Preterm <37	251 (11.4)	537 (12.1)	788 (11.9)
Term 37–41	1939 (87.8)	3870 (87.2)	5809 (87.4)
Postdates >41	18 (0.8)	33 (0.7)	51 (0.8)
**Antenatal visits at study hospital**			
0	62 (2.8)	163 (3.7)	225 (3.4)
1–3	1081 (49.0)	2360 (53.1)	3441 (51.7)
≥4	1065 (48.2)	1921 (43.2)	2986 (44.9)
**Number of fetuses in current pregnancy^a^**			
One	2176 (97.0)	4367 (96.5)	6543 (96.7)
Two	58 (2.6)	154 (3.4)	212 (3.1)
Three	9 (0.4)	3 (0.1)	12 (0.2)
**Number of previous spontaneous abortions**			
0	1778 (80.5)	2778 (62.5)	4556 (68.5)
1	259 (11.7)	992 (22.3)	1251 (18.8)
2–4	168 (7.5)	632 (14.3)	800 (12.0)
5–10	3 (0.1)	42 (0.9)	45 (0.7)
**Previous bad obstetric history**	32 (1.4)	335 (7.5)	368 (5.5)
**Number of previous caesarean sections** (multiparous only)			
1		449 (10.1)	
2		169 (3.8)	
3		63 (1.4)	
4		9 (0.2)	

a Included all newborns: single, twins, and triplets (n=6767).

### Characteristics of the hospital setting

The hospital is located on the outskirts of a large city in the Punjab. The hospital uses paper partogram (data not available electronically) to record fetal heart rate, maternal temperature, pulse and blood pressure, frequency and strength of contractions, cervical effacement and dilatation, color of amniotic fluid, and drugs administered. The hospital defines established labor as regular uterine contractions every 5 minutes lasting at least 40 seconds. The strength and length of the contractions is assessed by palpation. Vaginal examinations are performed on admission and every 4 hours in established labor. Slow progress in established labor is defined as no progress within 4 hours, primarily measured by vaginal dilatation. Epidural analgesia was not an option. Fetal heart monitoring is done using pinards. Women have a female relative with them at all times. A student midwife is by the side of a woman almost constantly, while staff midwives cover several students and women.

The hospital has one procedure for the administration of oxytocin, used for both induction and augmentation. Administration of oxytocin to women PROM is described as augmentation. Administration of oxytocin has to be prescribed by a doctor or senior midwife on duty. Administration of oxytocin started with 5 units of oxytocin in 1000 mL intravenous fluid, starting with 10 drops per minute and doubling the number of drops every 15 minutes, to a maximum of 40 drops/min. After 15 minutes with 40 drops/min, 5 units oxytocin was added in the same bag of IV fluids every 15 minutes until labor/regular strong contractions were established, or the maximum dose of 20 units oxytocin at 40 drops/min was reached. No electronic pumps are available for the titration of intravenous fluids. Oxytocin is usually continued until the third stage of labor is completed. Dosage is reduced if indicated by fetal or maternal response.

The study was approved by the Regional Ethical Committee South-Eastern Norway, Number 2018/516. Approval for the study was given from the board of the hospital and the ethical committee of the hospital in Pakistan. All data in the study were routinely collected and had no negative impact on the patients or the personnel at the hospital. Sensitive data were removed from the dataset and the data were anonymized prior to transfer to Norway for analysis.

### Statistical methods

The Statistical Package for the Social Sciences (SPSS, version 24) was used for the analysis and a p-value of 0.05 was used as a cut-off for significance. All women were included in the cross tabulations for Table 1. P-values were calculated using Pearson chi-squared test and Fisher’s exact test (<5 values in a cell). Elective CS and multiple pregnancies were excluded from the analyses on the use of oxytocin. Binary logistic regression analysis was used to estimate crude odds ratios (OR) and adjusted odds ratios (AOR) with 95 % confidence intervals (CI), investigating the association between the use of oxytocin and emergency CS. Adjustments were made for maternal age, gestational age, birthweight in two groups, bad obstetric history, presentation/lie, and previous CS for multiparous women.

## RESULTS

Of all 6652 woman who gave birth at the hospital in 2014 and 2015, 33.2% where primiparous and 66.8% were multiparous (Table 1). The mean maternal age for primiparous was 25.1 years (SD=3.7) and for multiparous 28.7 years (SD=4.6). Less than half of the women had ≥4 antenatal visits and almost 12% gave birth preterm (Table 1).

The overall CS rate was 15.0% with similar rates for primiparous and multiparous women but a different distribution for the proportion of elective CS and emergency CS (Table 2). There were no maternal deaths and no ruptured uterus in the study period. Of all babies, 130 were stillborn and 112 died before discharge, with no significant difference (p=0.36) between primiparous and multiparous. Primiparous women were significantly more likely to have an episiotomy compared to multiparous women, 60.6 % vs 2.0%, respectively (p<0.001) (Table 2).

**Table 2:** Obstetric and neonatal outcomes by parity for 2014 and 2015 (N=6652)

	*Primiparous women n=2208*	*Multiparous women n=4444*	*Total N=6652*
	*n (%)*	*n (%)*	*n (%)*
**Mode of delivery^a^**	n=2243	n=4524	n=6767
Spontaneous vaginal delivery	1768 (78.9)	3709 (81.9)	5477 (80.9)
Vaginal breech delivery	77 (3.4)	145 (3.2)	222 (3.3)
Emergency CS	324 (14.4)	302 (6.7)	626 (9.3)
Elective CS	27 (1.2)	361 (8.0)	388 (5.7)
Vaginal operative delivery (excluding breech)	47 (2.1)	7 (0.2)	54 (0.8)
**Indication for emergency CS**	n=324	n=302	n=626
No progress	230 (71.0)	148 (49.0)	378 (60.3)
Previous CS	0 (0.0)	22 (7.3)	22 (3.5)
Malpresentation	11 (3.4)	67 (22.2)	78 (12.5)
Fetal reason	52 (16.0)	23 (7.6)	75 (12.0)
Maternal reason	18 (5.6)	29 (9.6)	47 (7.5)
Not registered	13 (4.0)	13 (4.3)	26 (4.2)
**Birthweight** (g)^a^	n=2243	n=4524	n=6767
Low <2500	395 (17.6)	634 (14.0)	1029 (15.2)
Normal ≥2500	1848 (82.4)	3890 (86.0)	5738 (84.8)
**Neonatal outcome^a^**	n=2243	n=4524	n=6767
Alive	2156 (96.1)	4369 (96.6)	6525 (96.4)
Dead	87 (3.9)	155 (3.4)	242 (3.6)
Still birth	49 (56.3)	81 (52.3)	130 (53.7)
Neonatal death	38 (43.7)	74 (47.7)	112 (46.3)
**Gender^a^**	n=2243	n=4524	n=6767
Female	1122 (50.0)	2086 (46.1)	3208 (47.4)
Male	1121 (50.0)	2433 (53.8)	3554 (52.5)
Uncertain	0 (0.0)	5 (0.1)	5 (0.1)
**Presentation/lie^a^**	n=2243	n=4524	n=6767
Vertex	2110 (94.1)	4238 (93.7)	6348 (93.8)
Face	4 (0.1)	7 (0.2)	11 (0.1)
Breech	129 (5.8)	265 (5.9)	394 (5.8)
Transverse	0 (0.0)	14 (0.3)	14 (0.2)
PPH >1000 mL	19 (0.9)	38 (0.9)	57 (0.9)
**Episiotomy^b^**	1338 (61.3)	91 (2.2)	1429 (22.8)
**Tears in perineum^c^**			
Grade 1	78 (3.6)	182 (4.5)	260 (4.1)
Grade 2	27 (1.2)	50 (1.2)	77 (1.2)
Grade 3	0 (0.0)	4 (0.1)	4 (0.06)

a Included all newborns (n=6767). b Proportion calculated excluding elective caesarian section (CS). c Alone or in addition to an episiotomy, proportion excluding elective CS.

Of all women, 2347 (38.0%) were treated with oxytocin with primiparous women significantly more frequently receiving this treatment compared to multiparous, 60% vs 30.5%, respectively (Table 3). Almost all women induced received oxytocin (96.9%) while primiparous more frequently received misoprostol in addition (Table 3). Of the women with a spontaneous start of labor, including PROM, 1277 (25.7%) were augmented with oxytocin. Significantly more primiparous than multiparous women were augmented, 42.5% (749/1760) vs 16.1% (528/3277), respectively (p<0.001). Excluding women with PROM from spontaneous start of labor 16.5% (741/4481) were augmented, 13.8% started in first stage and 2.6% in second stage. Almost ten per cent (9.4%) of preterm births at the hospital were induced, most due to preeclampsia (29.7%), intrauterine growth restriction (24.7%), and stillbirths (21.6%) (data not in table).

**Table 3:** Status on admission, induction and augmentation in labor (N=6172)^a^

	*Primiparous Women n=2151*	*Multiparous Women n=4021*	*Total N=6172*	*p*
	*n (%)*	*n (%)*	*n (%)*	
**Status on admission**	n=2151	n=4021	N=6172	
Spontaneous start of labor^b^	1760 (81.8)	3277 (81.4)	5037 (81.6)	0.762
Admitted for induction	378 (17.6)	718 (17.9)	1096 (17.8)	
Missing (not elective CS)	13 (0.6)	26 (0.7)	33 (0.6)	
**Induction method**	n=378	n=718	n=1096	
Oxytocin only	136 (36.0)	418 (58.3)	554 (50.5)	<0.001
Misoprostol	10 (2.6)	19 (2.6)	29 (2.6)	
Misoprostol & oxytocin	231 (61.1)	277 (38.6)	508 (46.4)	
Balloon catheter	0 (0.0)	1 (0.1)	1 (0.1)	
Balloon catheter & oxytocin	1 (0.3)	3 (0.4)	4 (0.4)	
**Gestational age at induction** (weeks)	n=378	n=718	n=1096	
<37	25 (6.6)	47 (6.5)	72 (6.6)	0.360
37–41	341 (90.2)	656 (91.4)	997 (90.9)	
>41	12 (3.2)	13 (1.8)	25 (2.3)	
Not registered	0 (0.0)	2 (0.3)	2 (0.2)	
**Primary indication for induction**	n=378	n=718	n=1096	
Fetal reasons	257 (67.9)	261 (36.4)	518 (47.3)	<0.001
Maternal reasons	83 (22.0)	133 (18.5)	216 (19.7)	
Bad obstetric history	25 (6.6)	316 (44.0)	341 (31.1)	
Other	1 (0.3)	0 (0.0)	1 (0.1)	
Not registered	12 (3.2)	8 (1.1)	20 (1.8)	
**Augmentation of labor**	758 (35.2)	539 (13.4)	1297 (21.0)	<0.001
**Method and timing of augmentation**	n=758	n=539	n=1297	
Oxytocin after PROM^c^	246 (32.5)	195 (36.2)	441 (34.0)	0.360
Oxytocin in first stage of labor^c^	359 (47.3)	262 (48.7)	621 (47.9)	
Oxytocin in second stage of labor^c^	77 (10.2)	43 (8.0)	120 (9.3)	
Oxytocin & misoprostol after ruptured membranes	67 (8.8)	28 (5.1)	95 (7.3)	
Misoprostol after ruptured membranes	9 (1.2)	11 (2.0)	20 (1.5)	
**Primary indication for augmentation**	n=758	n=539	n=1297	
Ruptured membranes no contractions	322 (42.5)	234 (43.4)	556 (42.9)	0.415
Slow progress in Stage 1	359 (47.3)	261 (48.4)	620 (47.7)	
Slow progress in Stage 2	77 (10.2)	43 (8.0)	120 (9.3)	
Not registered	0 (0.0)	1 (0.2)	1 (0.1)	
**Infusion with oxytocin**	n=1117	n=1226	n=2343	<0.001
Induced	368 (33.0)	698 (56.9)	1066 (45.5)	
Augmented only	749 (67.0)	528 (43.1)	1277 (54.5)	

a Multiple pregnancies and elective CS excluded.

b Augmentation after Pre-labor Rupture of Membranes (PROM) is included in spontaneous start of labor as per hospital protocol.

c Oxytocin only.

Among primiparous women, the use of oxytocin for induction, PROM and in the first stage of labor was associated with an increased risk for emergency CS: (AOR=6.01; 95 %CI: 4.29–8.42), (AOR=2.43; 95% CI: 1.66– 3.56) and (AOR=1.80; 95% CI: 1.21–2.68), respectively (Table 4).

**Table 4:** Oxytocin and other associated factors with birth by emergency caesarean section in primiparous women (N=2151)^a^

	*Emergency CS n=316*	*Crude OR (95% CI)*	*AOR (95% CI)^b^*
Oxytocin only (n=309)^c^	n (%)		
**Use of oxytocin and reason**			
Ruptured membranes	57 (18.4)	3.29 (2.52–4.30)	2.43 (1.66–3.56)
Stage 1	47 (15.2)	2.09 (1.57–2.78)	1.80 (1.21–2.68)
Stage 2	6 (1.9)	1.70 (0.93–3.12)	0.95 (0.40–2.28)
Induced	123 (39.9)	4.04 (3.30–4.95)	6.01 (4.29–8.42)
No oxytocin	76 (24.6)	Ref.	Ref.
**Maternal age** (years)			
<25	118 (37.3)	0.65 (0.51–0.83)	0.81 (0.62–1.05)
25–37	195 (61.8)	Ref.	Ref.
≥38	3 (0.9)	4.86 (0.97–24.24)	2.87 (0.43–19.10)
**Gestational age** (weeks)			
<37	27 (8.5)	0.76 (0.50–1.16)	0.92 (0.58–1.47)
37–41	285 (90.2)	Ref.	Ref.
≥42	4 (1.3)	1.62 (0.53–4.97)	0.75 (0.23–2.46)
**Birthweight** (g)			
<3650	267 (84.5)	Ref.	Ref.
≥3650	49 (15.5)	3.60 (2.48–5.22)	3.57 (2.40–5.32)
**Bad obstetric history**			
Yes	11 (3.5)	3.12 (1.49–6.53)	1.05 (0.48–2.32)
No	305 (96.5)	Ref.	Ref.
**Presentation/lie**			
Vertex	282 (89.2)	Ref.	Ref.
No vertex	34 (10.8)	3.13 (2.04–4.82)	3.36 (2.07–5.46)

a Excluding multiple pregnancies and elective CS.

b Controlled for all variables in the table.

c Use of oxytocin not registered for 7 primiparous women. AOR: adjusted odds ratio.

Among multiparous women, only the use of oxytocin for induction and PROM was associated with an increased risk for emergency CS: (AOR=2.72; 95% CI: 1.78–4.13) and (AOR=2.05; 95% CI: 1.18–3.55), respectively (Table 5).

**Table 5:** Oxytocin and other associated factors and birth by emergency caesarean section in multiparous women (N=4021)^a^

	*Emergency CS n=273*	*Crude OR (95% CI)*	*AOR (95% CI)^b^*
Oxytocin only (n=254)^c^	n (%)		
**Use of oxytocin and reason**			
Ruptured membranes	22 (8.7)	2.12 (1.31–3.38)	2.05 (1.18–3.55)
Stage 1	20 (7.8)	1.59 (0.98–2.59)	1.27 (0.74–2.18)
Stage 2	4 (1.6)	1.98 (0.70–5.61)	2.33 (0.73–7.43)
Induced	71 (28.0)	2.18 (1.62–2.94)	2.72 (1.78–4.13)
No oxytocin	137 (53.9)	Ref.	Ref.
**Maternal age** ( years )			
<25	30 (11.0)	0.55 (0.37–0.82)	0.64 (0.41–1.01)
25–37	224 (82.0)	Ref.	Ref.
≥38	19 (7.0)	1.93 (1.17–3.19)	1.85 (1.02–3.34)
**Gestational age** (weeks) (n=272)			
<37	49 (18.0)	1.72 (1.25–2.39)	1.71 (1.14–2.56)
37–41	219 (80.5)	Ref.	Ref.
≥42	4 (1.5)	2.15 (0.75–6.17)	1.01 (0.29–3.52)
**Birthweight** (g)			
<3650	219 (80.2)	Ref.	Ref.
≥3650	54 (19.8)	1.81 (1.32–2.47)	2.12 (1.46–3.10)
**Bad obstetric history**			
Yes	29 (10.6)	1.37 (0.92–2.05)	1.13 (0.64–1.99)
No	244 (89.4)	Ref.	Ref.
**Previous caesarean section**			
≥1 previous CS	142 (52.1)	16.73 (12.74–21.97)	21.95 (16.10–29.94)
No	131 (47.9)	Ref.	Ref.
**Presentation/lie**			
Vertex	225 (82.4)	Ref.	Ref.
No vertex	48 (17.6)	6.24 (4.35–8.93)	7.54 (4.76–11.95)

a Excluding multiple pregnancies and elective CS.

b Controlled for all variables in the table.

c Use of oxytocin not registered for 19 multiparous women. AOR: adjusted odds ratio.

## DISCUSSION

The present study found a high standard of care at this hospital, as observed by the rate of spontaneous vaginal births and caesarean sections, perinatal mortality rate, and no cases of ruptured uterus or maternal mortalities. Vaginal breech delivery was common, while operative vaginal births were few. In particular, primiparous women were prone to receive interventions such as oxytocin and episiotomy.Oxytocin for induction had the strongest association with emergency CS, especially for primiparous women.Oxytocin for PROM was associated with double the odds of an emergency CS, for both primiparous and multiparous women. Oxytocin in the second stage of labor was not associated with an increased risk of an emergency CS.

We found the strongest association to be between the use of oxytocin for induction and emergency CS. This is not strange as inducing labor requires an indication. Usually, the indication in itself already increases the odds for a birth by CS^26^. We found that the adjusted odds of an emergency CS were six-fold for induced primiparous women. These increased odds are considerably larger than the doubling of the odds reported by the authors of a large cross-sectional study of routinely collected data in Australia of primiparous women at term^27^. However, this Australian study selected a ‘standard primipara’ excluding any complication or factors that could cause complications, which could explain some of the difference^27^.

Among the multiparous women there was a two-fold increase in odds for an emergency CS due to induction. This is in line with other retrospective studies, which consistently demonstrate a higher CS rate following induction^28^. In contrast, evidence from prospective studies suggests that compared to expectant management, induction of labor in women with intact membranes reduces the risk of CS^29^.

The hospital follows the WHO recommendations for induction of labor, including the need for a clear medical indication^17^. This may be reflected in the rate of induction in this study. Adding the number of women who received oxytocin for PROM to the number of women induced, as is common internationally, the proportion of induction was 23.1% at the study hospital. This is similar to the US with 23.8% in 2015^30^, and a little less than in England in 2014–2015^31^. Rates in low-and-middle-income countries vary,but are usually lower than in high-income countries^17^.

Among primiparous women augmentation with oxytocin in first stage of labor was associated with increased odds. Similar to induction, the reason for slow progress may become the reason for emergency CS. As oxytocin is a powerful drug with potential serious side-effects, minimizing its use is generally advised^32^. The rate of augmentation, excluding PROM, induction, multiple pregnancy and elective CS, was 16.6% (741/4481) for all, 10% for multiparous, and 29.9% for primiparous women. This is similar to the findings in the population-based large cohort study from England, the Birthplace study, which reported an incidence of 9.8/100 for multiparous women and 34/100 for nulliparous women planning to give birth in an obstetric unit^33^. In contrast to our study, the Birthplace study had a selected population of low-risk women. Judicious use of oxytocin has been associated with a reduction in emergency CS in several studies^32^ while other studies have found no increase in CS^34^. There were no cases of ruptured uterus among women receiving oxytocin, suggesting careful use of oxytocin at the study site, in particular when over half of the multiparous women had a previous CS.

The rate of episiotomies among primiparous women was considerably higher than the rates found in many high-income countries and recommendations found in the literature^35,36^. Probably, as a result, there were few 1st and 2nd degree tears (5.2 %). Cases of anal sphincter trauma were rare (0.1 %). While this might suggest that in this hospital the high rate of episiotomy prevents 3rd and 4th degree tears, it could also indicate that cases of anal sphincter trauma go undiagnosed. There were less than 1% vaginal operative deliveries performed during the study period. This may, in part, explain the extremely low prevalence of anal sphincter trauma. The proportion of vaginal operative deliveries varies greatly across countries and hospitals and appears to be declining across the globe^37,38^. Performing fewer operative deliveries poses a challenge, as staff will become less skilled, and confident and education opportunities decrease^38^. In contrast, there were a relative high number of vaginal breech deliveries during the study period, 222 (3.3%), a rate which is decreasing in high-income countries^39^.

### Strengths and limitations

A definite strength of the current study is the quality of the data used for analysis, except for PPH. The number of women registered with PPH >1000 mL of less than 1% is likely the result of underestimation and/or lack of reporting. The retrospective design meant that no Hawthorn effect has improved the findings due to increased focus. The study included a large number of women, which provided the opportunity to describe aspects of the obstetric care at the hospital and perform adjusted regression analyses. However, we only investigated data from 2 years and do not know if the rates of interventions in this hospital are developing over time. The registered data lacked information on socioeconomic background, education, body mass index (BMI) and obstetric outcomes such as Apgar score, which is a weakness of the study.

## CONCLUSIONS

The caesarean section rate of this hospital is in line with the recommendations of the WHO. This study found that the use of oxytocin during labor is associated with an increased risk for birth by emergency CS. Women who received oxytocin for induction had highest risk for emergency CS, in particular among primiparous women. This information can be used when advising women about induction. The findings of this study will stimulate continued careful recording and further and more detailed investigation of obstetric and midwifery practice at the study site.
